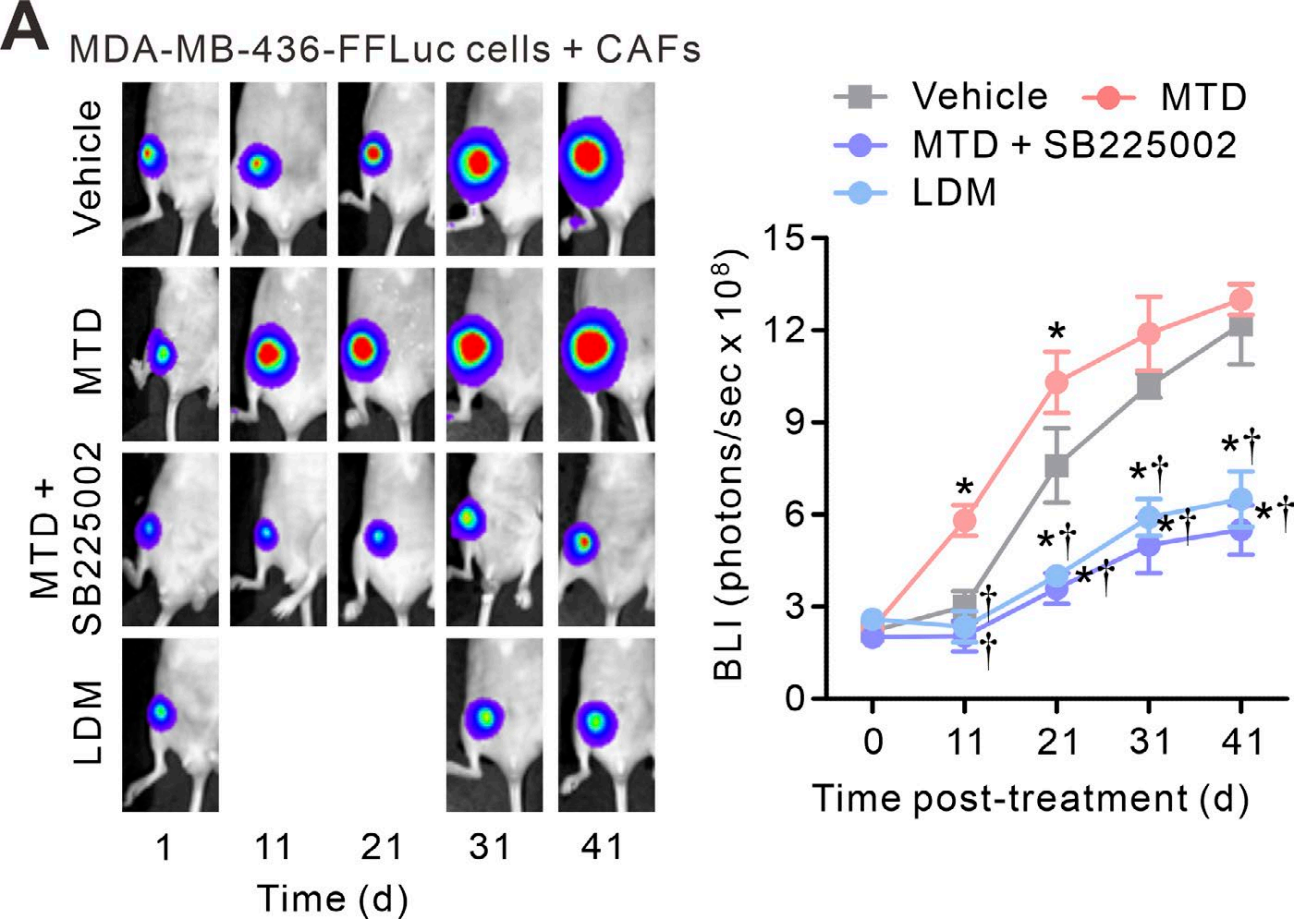# Correction: Metronomic chemotherapy prevents therapy-induced stromal activation and induction of tumor-initiating cells

**DOI:** 10.1084/jem.2015166506142023c

**Published:** 2023-06-20

**Authors:** Tze-Sian Chan, Chung-Chi Hsu, Vincent C. Pai, Wen-Ying Liao, Shenq-Shyang Huang, Kok-Tong Tan, Chia-Jui Yen, Shu-Ching Hsu, Wei-Yu Chen, Yan-Shen Shan, Chi-Rong Li, Michael T. Lee, Kuan-Ying Jiang, Jui-Mei Chu, Gi-Shih Lien, Valerie M. Weaver, Kelvin K. Tsai

Vol. 213, No. 13 | https://doi.org/10.1084/jem.20151665 | November 23, 2016

The bioluminescence images on days 11 and 21 of the “LDM” group in Fig. 8 A, which are provided as examples of tumor size on those days, are similar. The authors are certain that the two images are not the same; however, since the respective large-sized raw image files are no longer available, the authors cannot be certain that the images were collected at the time points listed. The two images have been removed from the corrected Fig. 8 A, shown here. The images in Fig. 8 A are representative only and do not impact the quantification and reproducibility of the data or the conclusions. The authors apologize for any confusion caused.

**Figure 8.**